# Caloric Restriction and Changes in Geroscience Blood-Based Biomarkers in Older Adults

**DOI:** 10.1093/geroni/igaf122.1020

**Published:** 2025-12-31

**Authors:** Fang-Chi Hsu, Rebecca Neiberg, Michael Miller, Philip Kramer, John Stone, Denise Houston, Barbara Nicklas, Stephen Kritchevsky

**Affiliations:** Wake Forest University School of Medicine, Winston-Salem, North Carolina, United States; Wake Forest University School of Medicine, Winston-Salem, North Carolina, United States; Wake Forest University School of Medicine, Winston-Salem, North Carolina, United States; Wake Forest University School of Medicine, Winston-Salem, North Carolina, United States; Wake Forest University School of Medicine, Winston-Salem, North Carolina, United States; Wake Forest University School of Medicine, Winston-Salem, North Carolina, United States; Wake Forest University School of Medicine, Winston-Salem, North Carolina, United States; Wake Forest University School of Medicine, Winston-Salem, North Carolina, United States

## Abstract

Intermediate endpoints are needed to evaluate the effect of interventions targeting the biology of aging in clinical trials. We examined associations between randomization to caloric restriction (CR), as well as achieved weight loss, and a consensus derived panel of blood-based geroscience biomarkers in 768 older adults (mean age, 67.4±5.2 years; 68% female and 81% Caucasian). Participants were randomly selected from six completed CR trials to provide a representative subset from each randomized group (CR vs. no CR) within each trial. Plasma CRP, IL-6, TNFr1, GDF15, cystatin C and insulin were measured at baseline and 6-months post-randomization to CR or no CR by the immunoassay platform Ella. Mean±SD weight loss over 6 months was 8.0±5.8 kg in the CR group and 1.4±3.7 kg in the no CR group. CR was associated with significant improvements in CRP and insulin compared to no CR (mean difference [95% CI]: -1.1 [-0.2, -1.9] mg/L and -15.3 [-8.1, -22.6] pmol/mL, respectively); however, there were no significant differences in IL-6, TNFr1, GDF15, or cystatin C by CR group. Achieved weight loss was associated with significant decreases in CRP, IL-6, and insulin (standardized betas (95% CI) per SD weight change: log transformed CRP: -0.26 (-0.13, -0.36), p < 0.0001; log transformed IL-6: -0.13 (-0.03, -0.23), p = 0.01; and insulin: -0.25 (-0.15, -0.36), p < 0.0001). We will also report the relationship between CR and a composite biomarker index. These results suggest that blood-based geroscience biomarkers are responsive to short-term CR, particularly those involved in inflammation and nutrient signaling.

